# Combined PCT, D-dimer and Fibrinogen in Paediatric Sepsis: Stratification Rather Than Diagnosis

**DOI:** 10.12669/pjms.41.12.13257

**Published:** 2025-12

**Authors:** Qing-Bao Jiang, Guo-Ming Zhang

**Affiliations:** 1 Qing-Bao Jiang, Department of Laboratory Medicine, Daqing Hospital of Traditional Chinese Medicine, Daqing 163311, Heilongjiang, People’s Republic of China; 2 Guo-Ming Zhang, Department of Laboratory Medicine, Shuyang Hospital, The Affiliated Shuyang Hospital of Xuzhou Medical University, Shuyang 223600, Jiangsu, People’s Republic of China

We read with great interest the article by Hao et al. on combined detection of procalcitonin (PCT), D-dimer (DD) and fibrinogen (FIB) in paediatric sepsis.^1^ While the findings suggest potential value, several methodological limitations restrict the strength of the conclusions.

First, the laboratory methodology requires clarification. The study employed a general biochemistry analyser for DD and FIB immunoturbidimetry but did not report kit manufacturers, detection limits or calibration data. Such omissions are important, as assay performance differs across platforms, especially in paediatric use.^2^

Second, the choice of control group is problematic. The “non-sepsis infected” controls already demonstrated increased inflammatory profiles (e.g., mean PCT >1 ng/L), which overlaps with the sepsis cohort. Without a healthy comparison, the results reflect stratification within infected patients rather than true diagnostic discrimination. The conclusion should therefore be limited to risk stratification, not diagnostic efficacy.^3^

Third, although C-reactive protein (CRP) correlated significantly with PCT in this study, CRP was excluded from the ROC analysis without justification. Considering that CRP remains widely used and that meta-analyses demonstrate its complementary role alongside PCT, omitting it weakens the evaluation of comparative diagnostic value.

Fourth, ROC curve reporting was insufficient. Although AUCs and confidence intervals were provided, no pairwise statistical comparisons were performed. Statements regarding the superiority of combined testing therefore lack statistical support.^2^

Finally, although patients were divided into mild and severe sepsis groups, no stratified ROC analyses were conducted to assess severity prediction. Group means were reported, but severity-specific diagnostic performance was not quantified, undermining the study’s claim to inform prognosis.

In summary, Hao et al. presented interesting preliminary data, but limitations in assay reporting, control group selection, biomarker inclusion, and ROC analysis restrict interpretation. We encourage the authors to reframe their findings as stratification evidence and to provide robust comparative analyses in future multicentre, prospective cohorts.

**Response from Authors**

***1:*** We acknowledge the reviewer’s concern regarding the need for detailed methodological reporting. In this study, the measurements of PCT, DD, and FIB were conducted using standardized commercial kits and automated analyzers following strict manufacturer instructions. PCT was determined using the MAGLUMI M11000 automatic chemiluminescence analyzer and its corresponding reagents. DD and FIB were detected using the Hitachi 7600 automatic biochemical analyzer and its associated immunoturbidimetry reagents. All procedures were performed in accordance with the clinical laboratory protocols of our institution. We recognize that providing detailed kit manufacturers, lot numbers, detection limits, and calibration data would enhance the reproducibility and transparency of our methods. We will ensure that such details are included in future studies to facilitate cross-platform comparisons and validation, particularly in pediatric populations.

***2:*** We thank the reviewer for this insightful observation regarding the control group. We acknowledge that the use of a “non-sepsis infected” control group, which exhibited elevated inflammatory markers (e.g., PCT >1 ng/L), means that our study design primarily assesses the ability of these biomarkers to stratify disease severity among children with infections. The clinical scenario we aimed to simulate is a common and critical challenge in pediatric emergency and critical care: distinguishing sepsis from other systemic infections in children who are already presenting with signs of inflammation. Our results demonstrate that PCT, DD, and FIB levels are significantly higher in children with sepsis compared to those with non-sepsis infections, and further increase with sepsis severity. Therefore, we agree with the reviewer that the primary value of our findings lies in risk stratification within a population of infected children. In our conclusion, the phrase “diagnostic value” is intended in this specific clinical context—differentiating sepsis from other infections and assessing its severity—rather than implying diagnosis from a healthy population. We affirm that the data support the conclusion that combined detection of these biomarkers is valuable for this stratification purpose, which is a crucial step in clinical management. We have taken care in our response to reframe the interpretation of our results to emphasize this point, and we agree that future studies including a healthy control group would be necessary to evaluate absolute diagnostic efficacy.

***3:*** We thank the reviewer for highlighting this point. Our correlation analysis did show a significant positive relationship between PCT and CRP (r=0.388, p=0.00). The primary focus of this study was to evaluate the relatively underexplored combination of PCT, DD, and FIB in pediatric sepsis. While CRP is a well-established inflammatory marker, it was not included in the ROC analysis due to the study’s specific aim. We acknowledge that incorporating CRP into the model could have provided a more comprehensive comparison. Future studies will include CRP and other conventional biomarkers in multivariate and ROC analyses to better evaluate their relative and combined diagnostic value.

***4:*** We thank the reviewer for raising this important methodological point. We acknowledge that the reporting of the ROC analysis would be strengthened by including formal statistical comparisons between the AUCs. In this study, the combined detection model yielded an AUC of 0.930, which is numerically superior to the AUCs of PCT (0.863), DD (0.831), and FIB (0.817) alone. While this numerical increase is substantial and clinically suggestive, we recognize that without direct pairwise statistical comparisons (e.g., using DeLong’s test), the statement of “superiority” in the conclusion is based on this observed difference rather than on a confirmed statistical significance. Therefore, we wish to clarify that the conclusion highlighting the higher diagnostic value of the combined detection reflects the compelling and consistent trend observed in our data. We agree that this finding should be interpreted as a strong preliminary indication, and we are committed to validating the statistical significance of this improvement in future prospective studies with a larger sample size, where such comparative analyses will be a key component of the statistical plan.

***5:*** We thank the reviewer for this valuable comment regarding the assessment of disease severity. We agree that performing stratified ROC analyses specifically for predicting the progression from mild to severe sepsis would provide a more direct and quantitative measure of prognostic performance. In this study, our primary analytical approach to inform prognosis was based on demonstrating statistically significant step-wise increases in the mean levels of PCT, DD, and FIB across control, mild sepsis, and severe sepsis groups, and by identifying these markers as independent related factors for sepsis through multivariate logistic regression. This methodology robustly establishes a strong association between the biomarker levels and disease severity. The conclusion that the combined detection has “clinical significance for determining the severity of the disease” is supported by this evidence of association and the model’s high overall accuracy in classifying the patient cohorts included in the study. We acknowledge that a formal severity-prediction analysis using stratified ROC curves was not conducted and that such an analysis would offer a more nuanced view of prognostic precision. We therefore interpret our current findings as demonstrating the utility of these biomarkers for severity assessment, and we fully agree with the reviewer that dedicated prognostic model development and validation, including the suggested stratified analyses, are a critical and necessary next step in future research.

***6:*** We sincerely thank the reviewer for their constructive summary. We agree that the methodological limitations identified—regarding assay reporting, control group selection, biomarker inclusion, and ROC analysis—necessitate a cautious interpretation of our results. Accordingly, we have reframed our findings to highlight their role in stratifying infection severity among children presenting with signs of infection, rather than asserting definitive diagnostic or standalone prognostic value. The promising performance of the combined biomarker model in this context justifies the future pathway recommended by the reviewer, and we are committed to validating these findings through a robust, multicenter prospective study that includes healthy controls, detailed assay standardization, direct comparisons with established biomarkers such as CRP, and advanced statistical analyses.

*Hongnian Duan*

*Email: duanhn2022@163.com*
